# The antiviral effects of baloxavir marboxil against influenza A virus infection in ferrets

**DOI:** 10.1111/irv.12760

**Published:** 2020-06-13

**Authors:** Mitsutaka Kitano, Takanobu Matsuzaki, Ryoko Oka, Kaoru Baba, Takahiro Noda, Yuki Yoshida, Kenji Sato, Kohei Kiyota, Tohru Mizutare, Ryu Yoshida, Akihiko Sato, Hiroshi Kamimori, Takao Shishido, Akira Naito

**Affiliations:** ^1^ Shionogi & Co., Ltd. Toyonaka Japan; ^2^ Shionogi TechnoAdvance Research, Co., Ltd. Toyonaka Japan

**Keywords:** baloxavir marboxil, ferrets, influenza A virus, pharmacodynamics, pharmacokinetics

## Abstract

**Background:**

Baloxavir marboxil (BXM), the oral prodrug of baloxavir acid (BXA), greatly reduces virus titers as well as influenza symptoms of uncomplicated influenza in patients.

**Objectives:**

To investigate the pharmacokinetic profiles of BXA and its efficacy against influenza A virus infection in ferrets.

**Methods:**

Ferrets were dosed orally with BXM (10 and 30 mg/kg twice daily for 1 day), oseltamivir phosphate (OSP) (5 mg/kg twice daily for 2 days) or vehicle to measure the antiviral effects of BXM and OSP. The pharmacokinetic parameters of BXA was determined after single oral dosing of BXM.

**Results:**

The maximum plasma concentrations of BXA were observed at 1.50 and 2.00 hours with the two BXM doses, which then declined with an elimination half‐life of 6.91 and 4.44 hours, respectively. BXM at both doses remained detectable in the plasma in ferrets, which may be due to higher stability in liver microsomes. BXM (10 and 30 mg/kg twice daily) treatment at Day 1 post‐infection (p.i.) reduced virus titers by ≥3 log10 of the 50% tissue culture infective doses by Day 2, which was significantly different compared with vehicle or OSP. Body temperature drops over time were significantly greater with BXM than with vehicle or OSP. Significant reduction in virus titers was also demonstrated when BXM was administrated after symptom onset at Day 2 p.i. compared with vehicle and OSP, although body temperature changes largely overlapped between Day 2 and Day 4.

**Conclusions:**

The results highlight the rapid antiviral action of BXM with post‐exposure prophylaxis or therapeutic dosing in ferrets and offer support for further research on prevention of influenza virus infection and transmission.

## 1 BACKGROUND

Seasonal influenza, an acute respiratory infectious disease caused by influenza viruses, spreads easily from person to person and causes seasonal epidemics worldwide.^1^, ^2^, ^3^, ^4^ Although influenza vaccination is the most effective way to prevent the disease, epidemics continue to occur, indicating the need for anti‐influenza drugs to control influenza virus infection.^5^, ^6^ Currently, neuraminidase inhibitors (NAIs), including oseltamivir phosphate (OSP), are the most widely used class of anti‐influenza drugs.^7^, ^8^

Baloxavir marboxil (BXM), and its active form baloxavir acid (BXA), is a first‐in‐class, small‐molecule inhibitor of cap‐dependent endonuclease (CEN),^9^ which has been approved for clinical use (single dose of 40 mg for patients 40 to <80 kg or 80 mg for patients ≥80 kg) in uncomplicated adults and adolescents in Japan and the United States for the treatment of influenza type A and B virus infections. The CEN in the polymerase acidic protein (PA), a subunit of influenza virus polymerase, mediates the cap‐snatching process during viral mRNA biosynthesis.^10^, ^11^ BXA exhibits broad‐spectrum inhibitory activity against seasonal, avian and swine influenza viruses including NAI‐resistant strains in vitro and in vivo.^9^, ^12^, ^13^

A previous phase 1 trial revealed that a single oral dose of BXM from 6 to 80 mg was rapidly metabolized to BXA, and the terminal elimination half‐life of BXA ranged from 49 to 91 hours.^14^ The phase 3 trial (CAPSTONE‐1) showed that infectious virus titers rapidly declined within 1 day after single, weight‐based oral doses of BMX (40 or 80 mg), and the average titer remained low thereafter, which were superior to both placebo and OSP.^15^ The time to alleviation of symptoms was also superior compared with placebo but not significantly different from the effect of OSP treatment. Treatment‐emergent amino acid substitution at amino acid position 38 in the PA (PA/I38T) was a major pathway for reduced susceptibility to BXA in the clinical study, as expected from non‐clinical evidence.^15^, ^16^

Ferrets are naturally susceptible to human influenza viruses without adaptation and develop upper respiratory tract infection.^17^, ^18^ The clinical signs of infected ferrets, including fever, nasal discharge, and decreased activity, closely reflect those manifested in humans.^19^ In addition, ferrets have the ability to transmit the influenza virus among each other.^20^, ^21^ Therefore, the ferret model is a useful tool for research on the evaluation of antivirals or vaccines as well as influenza virus transmission.^22^, ^23^, ^24^, ^25^, ^26^, ^27^

The aims of our study were to describe the pharmacokinetic profile and the antiviral effects of BXM against influenza A virus infection in ferrets to support our understanding of the pathogenesis of influenza.

## 2 METHODS

### 2.1 Compounds

Baloxavir marboxil and cefcapene pivoxil hydrochloride were synthesized by Shionogi & Co., Ltd. OSP was obtained from Sequoia Research Products Ltd. BXM was suspended in water containing 5% (w/v) sodium dodecyl sulfate (SDS) and 10% (w/w) polysorbate 80 (Wako Pure Chemical Industries) to improve oral absorption compared with 0.5% (w/v) methylcellulose solution (MC) (See Table S1, and Figure S1). OSP was dissolved in 0.5% MC.

### 2.2 Virus and cells

Influenza A virus A/Kadoma/3/2006 (H1N1) was kindly provided by Osaka Prefectural Institute of Public Health. Madin–Darby canine kidney (MDCK) cells were obtained from the European Collection of Cell Cultures and were maintained in minimum essential medium (MEM) supplemented with 10% fetal bovine serum (Invitrogen) and 100 μg/mL kanamycin sulfate (Invitrogen).

### 2.3 Animals

Female ferrets (Japan SLC Inc), confirmed to be negative for anti‐influenza antibodies, approximately 14‐26 months old were used. All ferret studies were conducted under applicable laws and guidelines and after approval from the Shionogi Animal Care and Use Committee. For stability tests, male Sprague Dawley (Crl:CD(SD)) rats and female cynomolgus monkey were obtained from Charles River Laboratories Japan, Inc. Male TOYO beagle was obtained from Oriental BioService, Inc.

### 2.4 Pharmacokinetic analysis

One day prior to administration, group assignment was carried out taking body weight into account. Under isoflurane anesthesia, fasted ferrets (four per group) received BXM (10 or 30 mg/5 mL/kg) suspension orally using an intragastric tube. Blood samples were collected at 0.5, 1, 2, 4, 6, 8, and 24 hours after single oral administration of BXM and centrifuged to obtain plasma. The concentrations of BXA and BXM in plasma were determined by liquid chromatography‐tandem mass spectrometry (LC‐MS/MS). The lower limit of quantification of BXA and BXM in ferret plasma was 0.5 ng/mL. The pharmacokinetic parameters of BXA and BXM were calculated using non‐compartmental analysis module in Phoenix WinNonlin software (version 8.0; Certara LP) (https://onlinehelp.certara.com/phoenix/8.2/topics/nca.htm) based on a non‐compartment model analysis with uniform weighting.

Plasma concentrations of BXA after dosing of BXM twice daily were estimated using the non‐parametric superposition function in Phoenix WinNonlin software (https://onlinehelp.certara.com/phoenix/8.2/topics/nonparasuper.htm) based on the mean plasma concentrations of BXA following single‐dose administration of BXM. Dosing type was used as “Variable.” The computation method used for interpolation and extrapolation of untransformed data was selected as logarithm (Log). The terminal elimination half‐life for 10 and 30 mg/kg doses was extrapolated from changes of the mean plasma concentrations of BXA in four ferrets between 6 and 24 hours, with values of 6.57 and 4.08 hours, respectively.

### 2.5 Stability in plasma

Approximately 30 mL of blood from Japanese male healthy volunteers was collected and centrifuged to obtain plasma. Blood samples from ferrets, rats, dogs, and monkeys were collected under anesthesia and centrifuged to obtain plasma. BXM was added to reach a final concentration of 200 ng/mL to all pooled plasma samples per species. After incubation for 5, 15, and 30 minutes at 37°C, twofold volume of ice‐cold acetonitrile/methanol (1:1, v/v) was added to the plasma samples to stop further reactions, which were then centrifuged at 1800 *g* for 10 minutes prior to LC‐MS/MS analysis of BXM in the supernatants.

The investigation followed the principles of the Declaration of Helsinki and governmental guidelines and was approved by the Ethics Committee on Human Tissue and Genome Research of Shionogi. Informed consent was obtained from all volunteer donors for use of the blood specimens in this study.

### 2.6 Metabolic stability in liver microsomes

Liver microsomes were prepared from ferrets, rats, dogs, and monkeys, as previously described.^28^ Human liver microsomes were obtained from Sekisui‐XenoTech, LLC and pooled (n = 15). Metabolic stability of the compounds in liver microsomes was tested in duplicate. Oseltamivir and cefcapene pivoxil were used as reference compounds. Liver microsomes at 0.5 mg/mL concentration (final volume of 0.2 mL) were incubated in Tris‐HCl buffer (pH 7.4), containing 50 mmol/L Tris‐HCl, 150 mmol/L KCl, 10 mmol/L MgCl_2_. The hydrolysis reaction was initiated by applying the test compound solution (final concentration 2 μmol/L). After incubation for 10, 20, and 30 minutes at 37°C, the hydrolysis was terminated by the addition of twofold volume of ice‐cold acetonitrile/methanol (1:1, v/v). Samples were centrifuged at 3000 rpm for 10 minutes prior to LC‐MS/MS analysis of compounds in the supernatants.

### 2.7 Virus infection and drug administration

#### 2.7.1 Experiment 1

Ferrets were anesthetized by a zolazepam, tiletamine, and xylazine mixture given intramuscularly and then intranasally inoculated with A/Kadoma/3/2006 (1000 tissue culture infective dose [TCID_50_]) in 200 μL of Dulbecco's phosphate‐buffered saline (DPBS). On Day 1 post‐infection (p.i.), fasted ferrets (four per group) were given oral BXM (10 or 30 mg/kg twice daily for 1 day), OSP (5 mg/kg twice daily for 2 days), or vehicle (water containing 5% SDS and 10% Tween 80) twice daily for 1 day (12‐hour interval between each dosing) (Figure 3A).

#### 2.7.2 Experiment 2

Ferrets were anesthetized as above and then intranasally inoculated with A/Kadoma/3/2006 (5000 TCID_50_) in 200 μL of DPBS. On Day 2 p.i., fasted ferrets (six per group) were given oral BXM (10 mg/kg twice daily for 1 day), OSP (5 mg/kg twice daily for 2 days), or vehicle (water containing 5% SDS and 10% Tween 80 twice daily for 1 day) (Figure 4A).

### 2.8 Analysis of virus titer in nasal washes and body temperature in ferrets

To monitor viral replication in nasal cavities, ferrets were lightly anesthetized with isoflurane and nasal wash fluids were obtained from each ferret using a disposable syringe filled with 5 mL of DPBS including penicillin and streptomycin on Days 1, 2, and 3 (Experiment 1), or Days 1, 2, 3, and 4 p.i. (Experiment 2). Nasal wash fluids were collected and filtered using 0.45 µm filter and then stored until use. For virus titration, serial dilutions of nasal washes were inoculated onto confluent MDCK cells in 96‐well plates. After 1 hour incubation, the suspension was removed and the cells were cultured in MEM including 0.5% bovine serum albumin (Sigma‐Aldrich) and 3 µg/mL trypsin. The plates were incubated at 37°C in 5% CO_2_ for 3 days. The presence of cytopathic effects was determined under a microscope, and virus titers were calculated as log_10_ TCID_50_/mL. When no cytopathic effect was observed using undiluted viral solution, it was defined as an undetectable level, being lower than 0.5 log_10_ TCID_50_/mL.

Under anesthesia, a data logger (DS1921H‐5F; Maxim Integrated Products, Inc) was implanted for subcutaneous body temperature monitoring. Penicillin and streptomycin were given to each ferret once daily for 3 days following surgery. Body temperature was analyzed using One Wire Viewer version 0.3.15.50. (Maxim Integrated Products, Inc) and expressed by calculating the average temperatures for 8‐hour periods. Data recorded from 8 hours prior to the first sampling of nasal wash were defined as the basal body temperature.

### 2.9 Statistical analysis

The uniformity of the mean body weight among groups was confirmed by one‐way analysis of variance (ANOVA). For the comparison of the virus titers in nasal washes and change from baseline body temperature among BXM‐, OSP‐, and vehicle‐treated groups, the fixed‐sequence procedure or Dunnett's multiple‐comparison method following the ANOVA test. *P* values of <.05 were considered to be statistically significant. Statistical analysis was performed using the statistical analysis software SAS version 9.2 (SAS Institute Inc).

## 3 RESULTS

### 3.1 Pharmacokinetic analysis of BXA and BXM in ferrets

**FIGURE 1:**
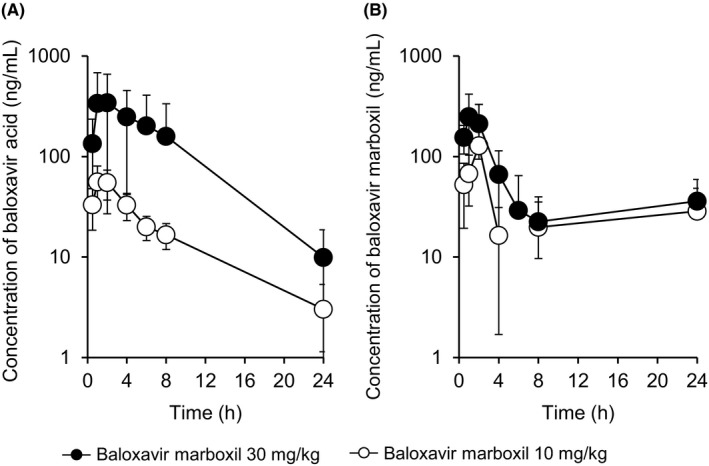
Plasma concentration‐time profiles of baloxavir acid (A) and baloxavir marboxil (BXM) (B) in ferrets after a single oral administration of BXM. Each plot represents mean ± standard deviations of four ferrets

The plasma exposures of BXA after a single oral administration of BXM at doses of 10 and 30 mg/kg are shown in Figure 1A. The maximum plasma concentrations of BXA were reached at 1.50 and 2.00 hours at doses of BXM 10 and 30 mg/kg, respectively and then declined with a mean (standard deviation) elimination half‐life (*t*_1/2_) values of 6.91 (3.79) and 4.44 (0.67) hours, respectively (Table 1 and Table S2). BXA was still detectable at 24 hours (3.03 ± 2.33 and 9.91 ± 8.77 ng/mL at doses of BXM 10 and 30 mg/kg, respectively). BXM was also detected in ferret plasma after a single oral administration of BXM at both doses (Figure 1B and Table 1).

**TABLE 1:** Pharmacokinetic parameters of baloxavir acid (BXA) and baloxavir marboxil (BXM) in ferrets after a single oral administration of BXM at doses of 10 and 30 mg/kg

Pharmacokinetic parameters	BXA		BXM	
	10 mg/kg	30 mg/kg	10 mg/kg	30 mg/kg
*C*_max_ (ng/mL)	66.6 ± 14.6	365 ± 316	165 ± 179	298 ± 88
*C*_max_ (nmol/L)	138 ± 30	755 ± 653	289 ± 312	522 ± 154
*C*_24 h_ (ng/mL)	3.03 ± 2.33	9.91 ± 8.77	28.6 ± 30.4	36.1 ± 12.3
*C*_24 h_ (nmol/L)	6.26 ± 4.82	20.5 ± 18.2	50.1 ± 53.3	63.1 ± 21.5
AUC_0‐24 h_ (ng·h/mL)	421 ± 119	3240 ± 3240	715 ± 758	1260 ± 150
AUC_0‐24 h_ (nmol/L ·h)	871 ± 246	6710 ± 6710	1250 ± 1330	2210 ± 250
*T*_max_ (h)	1.50 ± 0.58	2.00 ± 1.41	1.13 ± 0.63	0.875 ± 0.250
*T*_1/2_ (h)	6.91 ± 3.79	4.44 ± 0.67	NC	NC

Note

Data are expressed as the mean ± standard deviation of four ferrets. Individual values are described in Table S2.

Abbreviations: AUC_0‐24 h_, area under the concentration‐time curve from time 0‐24 h post‐dose; *C*_24 h_, concentration at 24 h; *C*_max_, maximum concentration; NC, not calculated; *T*_1/2_, terminal elimination half‐life; *T*_max_, time to maximum concentration.

**FIGURE 2:**
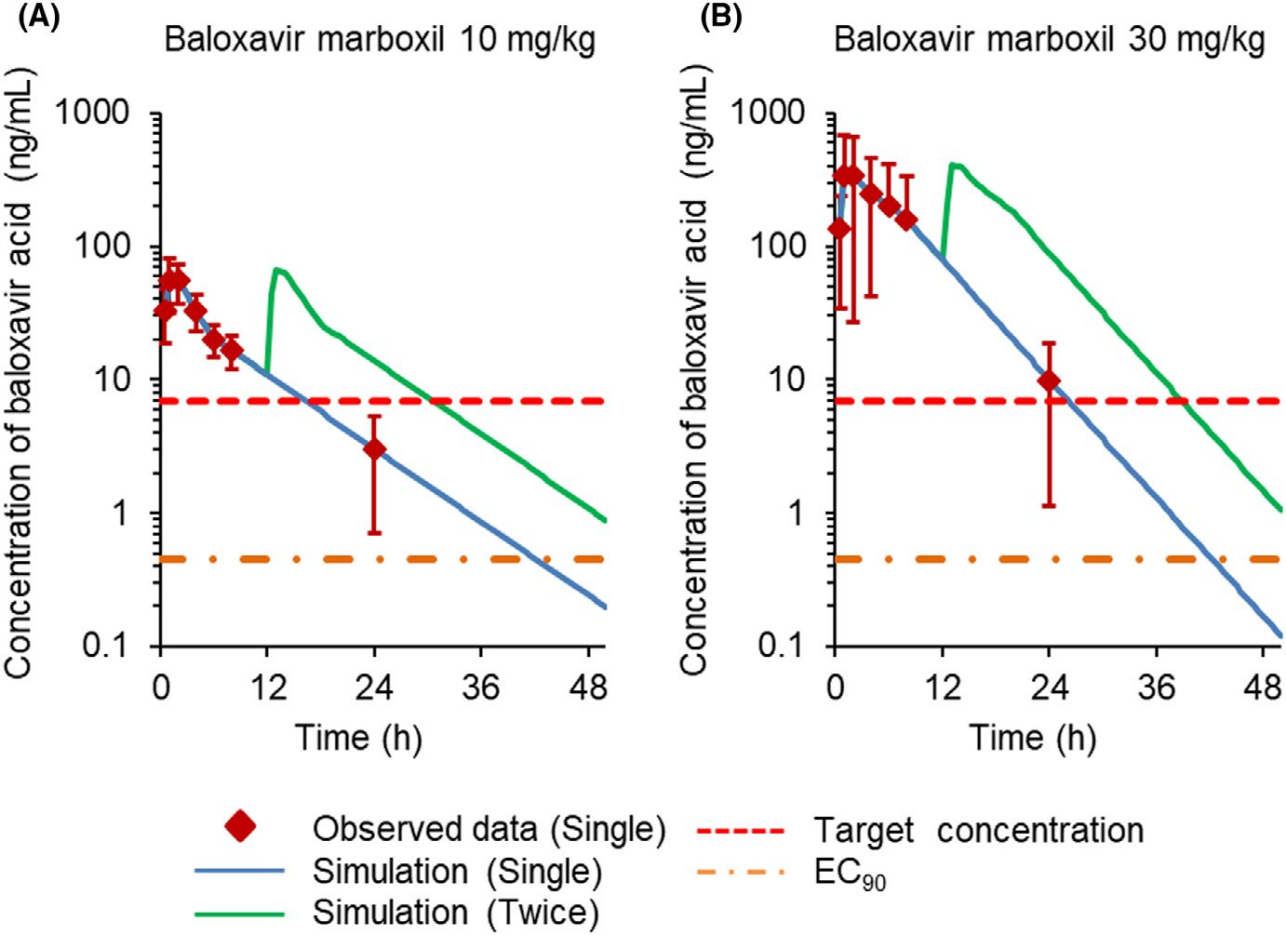
Simulated plasma concentration‐time profile of baloxavir acid (BXA) after oral administration of baloxavir marboxil twice a day on the first day. Each symbol indicates observed data for a single administration. The plasma BXA concentrations were estimated by computing the superposition from observed single‐dosing data in Phoenix WinNonlin Software^®^. The simulated values of plasma concentration are described in Table S3. The 90% effective concentration (EC_90_) value of BXA against A/Kadoma/3/2006 of 0.88 nmol/L (0.42 ng/mL) was based on a previous in vitro study.^9^ The target plasma concentration of BXA (6.85 ng/mL) was determined in a previous mouse study^36^

Due to a relatively short half‐life in ferrets, the plasma concentration changes of BXA over time were simulated for twice‐daily administration of BXM vs once‐daily administration (Figure 2). At 24 hours, the simulated plasma concentrations of BXA at both doses were much greater with twice‐daily administration than with once‐daily administration of oral BXM at doses of 10 and 30 mg/kg and remained above the target therapeutic level (Figure 2, and Table S3).

### 3.2 Stability of BXM in plasma and liver microsomes

The stability of BXM in plasma of humans and animals (except rats) was comparable (Table 2 and Table S4). We also compared the stability of BXM with that of OSP and cefcapene pivoxil, a reference prodrug to confirm hydrolytic activity, in liver microsomes of humans, ferrets, rats, dogs, and monkeys. At 30 minutes, cefcapene pivoxil was undetectable in liver microsomes of all species. OSP was stable in liver microsomes of ferrets, rats, and dogs, and BXM was stable only in ferret liver microsomes (Table 3, and Table S5).

**TABLE 2:** Stability of baloxavir marboxil (BXM) in plasma of a human, ferret, rat, dog, and monkey

Incubation time (min)	% Residual in plasma									
	Human		Ferret		Rat		Dog		Monkey	
	BXM	BXA^a^	BXM	BXA^a^	BXM	BXA^a^	BXM	BXA^a^	BXM	BXA^a^
10	92.3	8.3	94.7	4.6	0.0	104.6	91.6	2.8	82.9	9.0
20	78.6	22.4	90.4	13.9	0.0	104.0	79.7	8.2	71.8	24.0
30	63.9	38.1	77.4	24.9	0.0	103.2	75.6	15.6	55.5	43.0

Note

Data are expressed as the mean of duplicate. Individual values are described in Table S4.

^a^ % Residual of baloxavir acid (BXA) was calculated from BXM concentration at 0 min as molar concentration.

**TABLE 3:** Stability of baloxavir marboxil (BXM), oseltamivir, and cefcapene pivoxil in liver microsomes of a human, ferret, rat, dog, and monkey

Substance	Incubation time (min)	% Residual in liver microsome					
		Human	Ferret	Rat	Dog	Monkey	Control (Microsome free)
BXM	10	8.2	98.5	25.3	0.1	0.0	81.3
	20	0.3	98.1	4.6	0.1	0.0	81.7
	30	0.0	90.8	0.6	0.0	0.0	92.1
Oseltamivir	10	89.8	106.5	110.8	115.8	73.6	106.4
	20	64.8	107.2	119.2	109.6	59.1	105.4
	30	49.7	109.0	117.3	110.5	45.2	111.3
Cefcapene pivoxil	10	2.1	3.2	21.6	5.4	0.0	79.3
	20	0.0	0.1	1.6	0.2	0.0	67.7
	30	0.0	0.0	0.1	0.0	0.0	65.4

Note

Data are expressed as the mean of duplicates. Individual values are described in Table S5.

### 3.3 Effects of oral BXM when administered at Day 1 p.i. in ferrets

**FIGURE 3:**
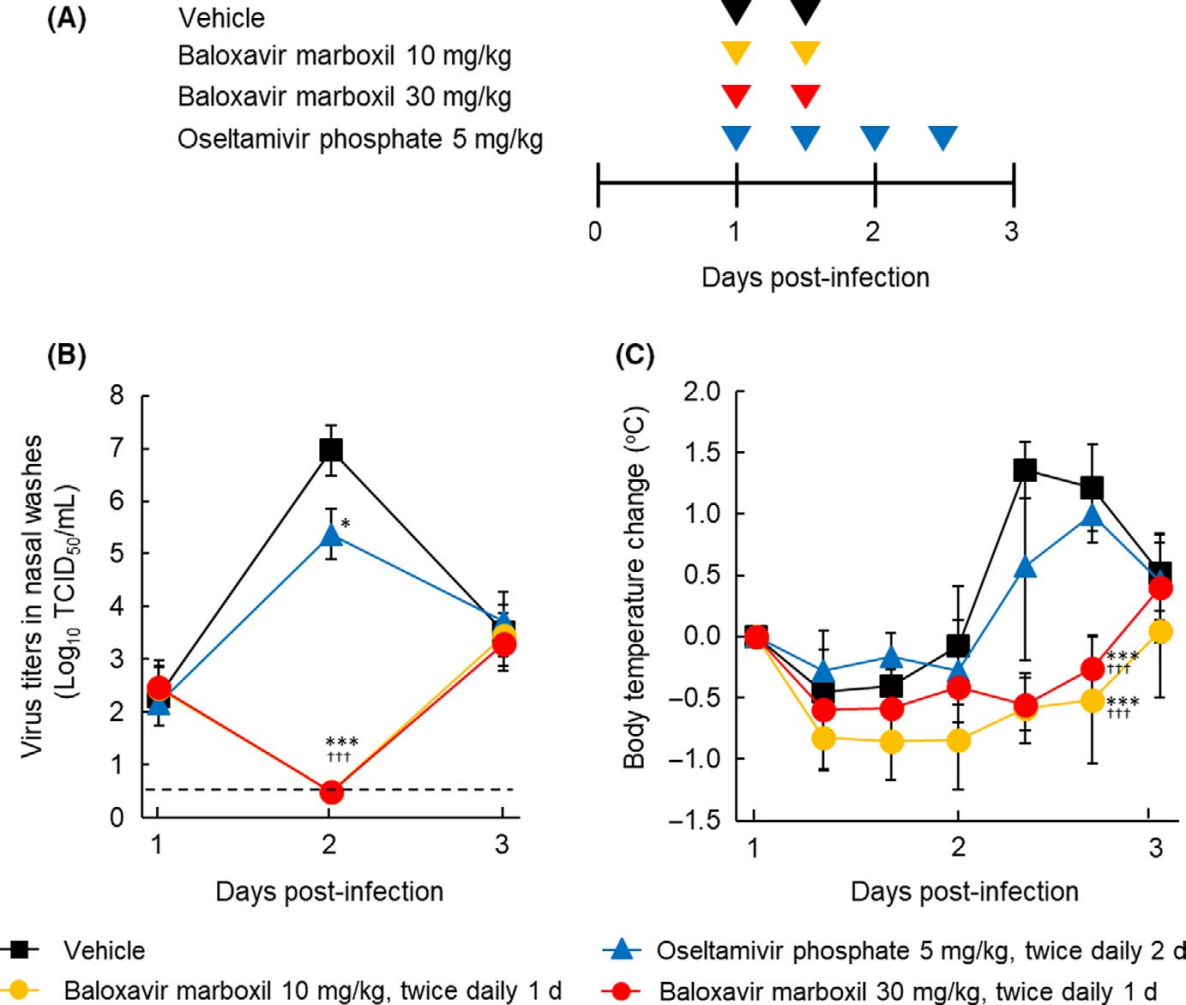
Virus titers and change in body temperature of infected ferrets treated with baloxavir marboxil or oseltamivir phosphate (OSP), starting at Day 1 post‐infection. Each plot represents the mean ± standard deviation of four ferrets. A, Dosing schedule for the efficacy study. B, Virus titers in nasal washes over time. C, Body temperature changes from baseline over time. TCID_50_, 50% tissue culture infective dose. Asterisks indicate statistically significant difference, as follows: *P < .01; ***P < .0001 compared with vehicle; ^†††^ *P* < .0001 compared with OSP

The maximum virus titer in nasal washes in vehicle‐treated ferrets was detected on Day 2 p.i., followed by a decline by Day 3 p.i. (Figure 3B). BXM at doses of 10 and 30 mg/kg showed a similar reduction in virus titer to an undetectable level (ie, <0.5 log_10_ TCID_50_/mL) at Day 2 p.i. OSP treatment at 5 mg/kg dose for 2 days resulted in a reduced burden of virus replication compared with vehicle. Virus titer levels were comparable by Day 3 among all groups, following a rebound in the BXM group (Figure 3B). A rise in body temperature from baseline was observed in the vehicle‐ and OSP‐treated groups from Day 2 to Day 3 p.i. (Figure 3C and Figure S2). The suppression of body temperature changes over time from 8 hours up to Day 3 p.i. was significantly greater with BXM at doses of 10 and 30 mg/kg than vehicle and OSP 5 mg/kg.

### 3.4 Effects of oral BXM when administered at Day 2 p.i. in ferrets

**FIGURE 4:**
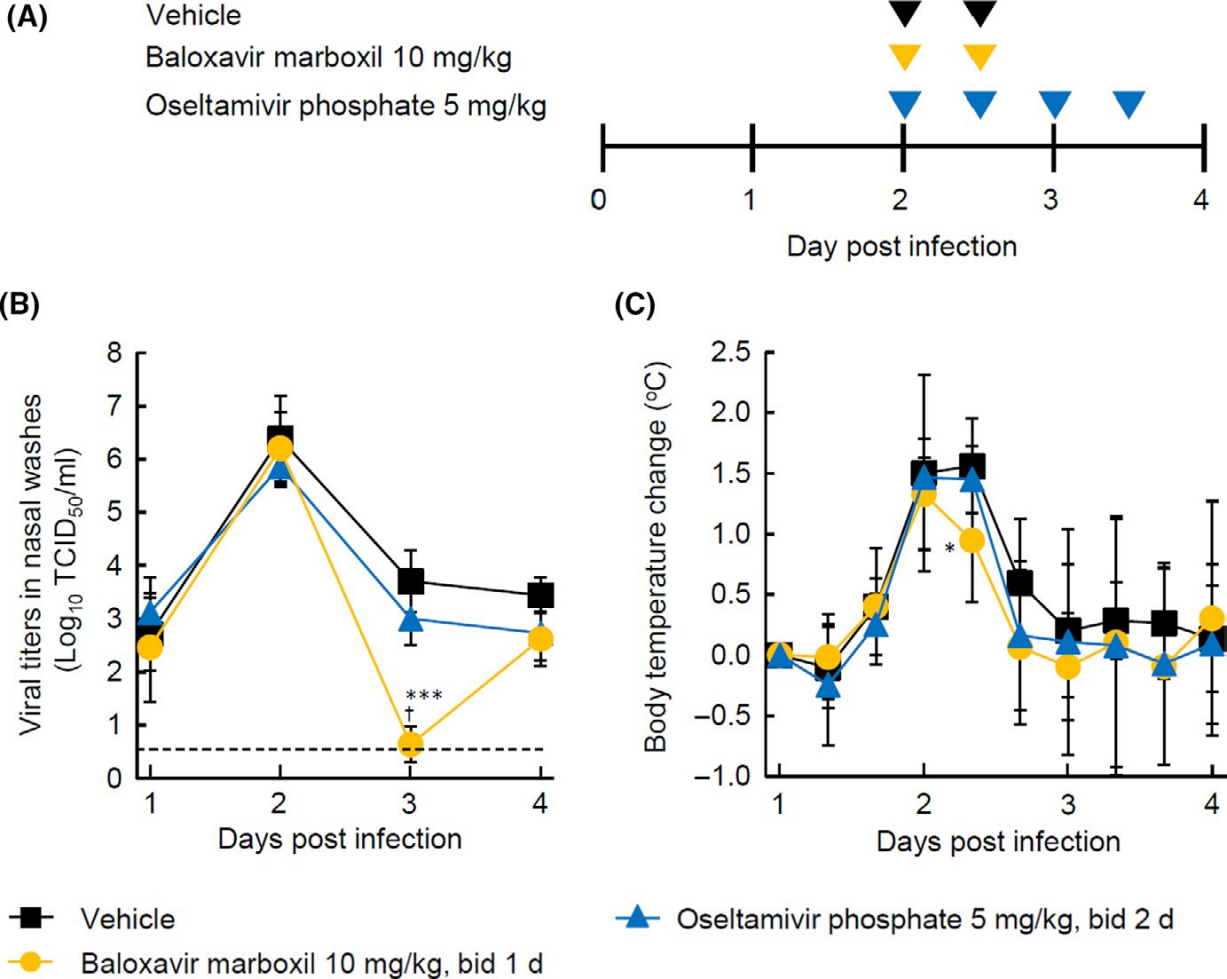
Virus titers and change in body temperature of infected ferrets treated with baloxavir marboxil or oseltamivir phosphate (OSP), starting at Day 2 post‐infection. Each plot represents the mean ± standard deviation of six ferrets. A, Dosing schedule for the efficacy study. B, Virus titers in nasal washes over time. C, Body temperature changes from baseline over time. TCID_50_, 50% tissue culture infective dose. Asterisks indicate statistically significant difference, as follows: *P < .05; ***P < .001 compared with vehicle; ^†^ *P* < .01 compared with OSP

In the second part of the experiments, ferrets were infected with influenza A/Kadoma/3/2006 (H1N1) strain intranasally at 5000 TCID_50_/ferret, which was a fivefold higher infectious dose increasing the body temperature by >1° by Day 2 p.i. BXM treatment at Day 2 for 1 day at the dose of 10 mg/kg resulted in a statistically significant reduction in virus titer by Day 3 compared with vehicle or OSP at the dose of 5 mg/kg twice daily (Figure 4B). OSP treatment also reduced virus titers on Day 3 p.i., but not significantly compared with vehicle. At Day 4, virus titers in nasal washes between BXM and OSP groups were similar (Figure 4B). The changes in body temperature over time were relatively similar between vehicle, OSP, and BXM treatment groups, although a small but significant effect was observed with BXM compared with vehicle on Day 2 (Figure 4C and Figure S3).

## 4 DISCUSSION

In this study, we examined the antiviral effects of BXM against influenza A virus infection in the ferret model. This is the first study to examine the pharmacokinetics and pharmacodynamics of a CEN inhibitor in this animal model. We previously reported the antiviral activities of BXA in vitro and in a mouse model.^9^, ^29^, ^30^, ^31^ Interestingly, the half‐life of BXA in ferrets was significantly shorter than in humans, suggesting that the plasma concentrations of BXA at 24 hours would be insufficient following single‐dose administration of 10 or 30 mg/kg. Therefore, plasma concentrations of BXA over 24 hours following twice‐daily oral dosing of BXM were simulated to describe comparable exposures in humans. In the clinical phase 2 study, administration of BXM at 10 mg dose resulted in a strong antiviral effect against influenza virus A compared with the placebo group.^32^ The plasma concentrations of BXA at 24 hours after BXM 10 or 40 mg dosing was 15.1 (range 6.38‐29.0) ng/mL or 61.5 (range 27.9‐118) ng/mL in Japanese patients, which was comparable to those in ferrets after 10 or 30 mg twice dosing (See Figure S4). Although high levels of BXM were detected in ferrets in contrast to humans and mice, its CEN inhibitory activity was ≥200‐fold lower than that of BXA in in vitro studies, which indicates that the plasma concentration of BXA is more crucial for determining the dosing regimen of BXM.^9^ Metabolic stability tests showed that BXM was stable in liver microsomes of ferrets, but not in any other species. BXM is metabolized by arylacetamide deacetylase (AADAC), which is expressed in the liver and the intestine in humans.^33^, ^34^ However, the tissue distribution of ferret AADAC is currently unknown. The species differences in AADAC expression or activity in liver may contribute to these findings.

Owing to the similarities in the pathogenesis of influenza in humans, ferrets are frequently used to describe antiviral activities of agents or to develop vaccines.^27^ In this study, we have assessed the antiviral effects of BXM in ferrets infected by influenza virus A/Kadoma/3/2006 (H1N1), which showed the highest virus titer in nasal washes among the virus strains we examined (M. Kitano, K. Baba, T. Noda, R. Yoshida, A. Sato, T. Shishido, & A. Naito, unpublished data). Our previous in vitro studies showed that the mean 90% effective concentration (EC_90_) value of BXA against influenza A/Kadoma/3/2006 strain in a yield reduction assay was 0.88 ± 0.49 nmol/L (0.42 ± 0.23 ng/mL), which was within the range of the EC_90_ values of BXA (0.63 to 0.95 nmol/L) obtained for other influenza A virus clinical strains.^9^ When the treatment for infected ferrets was started at Day 1 p.i., BXM at doses of 10 and 30 mg/kg was more effective for reduction of virus titers than vehicle or OSP at the dose of 5 mg/kg, and body temperature changes were also suppressed. Although treatment with OSP at the dose of 5 mg/kg significantly reduced virus titers compared with vehicle, no effect on body temperature was observed. These results indicate that potent suppression of virus replication by BXM was critical in achieving significant control of body temperature in ferrets, in contrast to suppression of viral shedding by OSP, which had no effect on changes in body temperature. Between groups treated with BXM at doses of 10 and 30 mg/kg, there were no significant differences in reduction of virus titers or body temperature changes, suggesting that BXM at the dose of 10 mg/kg achieved the maximum effects against influenza A/Kadoma/3/2006 strain. Even when BXM 10 mg/kg treatment was initiated at Day 2 p.i., the virus titers over time from Day 2 to Day 3 p.i. were significantly lower than those for vehicle or OSP. Following treatment at Day 2, when virus titers were very high, within 8 hours a small but significant drop in body temperature was seen with BXM compared with vehicle. Subsequent reductions in body temperature were not different between treatment groups and this paralleled reductions in virus titers. These results also indicate that a rapid suppression of virus replication at peak levels may explain the significant drop in body temperature in ferrets even when treatment was initiated after symptom onset. Although there was no significant difference in the time to alleviation of symptoms between BXM and OSP groups in uncomplicated influenza in humans,^15^ it might be due to differences between experimentally naïve ferrets and humans in immunity against the infection caused by the influenza virus. It has been suggested that significant improvement of symptoms by BXM could be observed in humans who have not been previously exposed to influenza virus (eg, infants).

There was an increase in virus titers when BXM treatment was stopped on Day 1, with titers reaching levels similar to those in vehicle‐ and OSP‐treated ferrets within 2 days post‐dosing. The increased virus titer was not associated with amino acid substitutions for reduced susceptibility to BXA although in humans PA/I38T was first detected at 2 days after dosing with BXM.^35^ The rebound in virus titer observed in the current study might be explained by insufficient plasma concentration of BXA in ferrets. The concentration of BXA decreased from 13.9 to 1.10 ng/mL between Day 1 and Day 2, which may explain the reduced antiviral effect. In addition, the simulated BXA concentrations at 48 hours were lower than the plasma target concentration (6.85 ng/mL) (see Table S6).^36^ Further studies for longer treatment periods that maintain the effective plasma concentration of BXA are required to examine the suppression of influenza virus replication continuously in vivo in ferrets. The complex and temporal humoral response mediated by B cells via the synthesis of specific antibodies peaks around 7‐14 days p.i. in response to seasonal influenza virus as explained by Lam and Baumgarth.^37^ We postulate that in the absence of influenza virus‐specific antibodies that would prevent the rebound of virus titer, it is necessary to maintain effective BXA concentrations in plasma until up to Day 7 p.i. or later in ferrets.^38^, ^39^

Laboratory strains with the I38T substitution in PA that confer reduced susceptibility to BXA showed reduced replication fitness in canine and human cells.^12^ Therefore, we postulated the possibility that variant viruses with similarly reduced replication fitness might have emerged in ferrets. In our non‐clinical and clinical resistance analysis based on Sanger sequencing method, we identified PA/I38 substitutions as a major pathway for resistance.^9^, ^12^, ^16^ In addition, using next‐generation sequencing of swab samples obtained from patients, novel types of treatment‐emergent amino acid substitutions were not identified.^35^, ^40^ Based on these findings, in order to detect the emergence of resistant variants after treatment with BXM or OSP, we analyzed PA and neuramidase (NA) gene sequences of the viruses obtained from nasal washes at Day 2 and Day 4 p.i. by the Sanger method, and we found no changes in PA or NA amino acids (data not shown). However, there are reports describing that variants with reduced susceptibility to baloxavir are fit and transmissible in ferrets.^40^, ^41^ Of note, treatment of immunocompromised ferrets with OSP led to mutant variants^42^, ^43^; thus, investigation of the antiviral effects of BXM in immunocompromised hosts (either ferrets or humans) would provide additional information for the treatment of high‐risk influenza patients.

In conclusion, we demonstrated that a single‐day oral dose of BXM showed the reduction in virus titers and symptoms on 1 day after administration in ferrets infected with influenza A virus. Furthermore, these effects observed with BXM were superior to those observed with OSP in ferrets. These results highlight the magnitude and rapidity of the antiviral effects of BXM against influenza A virus infection, which have been observed in clinical studies.

## CONFLICT OF INTEREST

The authors are employees of Shionogi and Co., Ltd. Osaka, Japan.

## AUTHOR CONTRIBUTION

**Mitsutaka Kitano:** Conceptualization (lead); Data curation (lead); Formal analysis (lead); Investigation (lead); Methodology (lead); Project administration (lead); Resources (lead); Software (lead); Supervision (lead); Validation (lead); Visualization (lead); Writing‐original draft (lead); Writing‐review & editing (lead). **Takanobu Matsuzaki:** Conceptualization (lead); Data curation (lead); Formal analysis (lead); Investigation (lead); Methodology (lead); Software (lead); Supervision (lead); Validation (lead); Visualization (lead); Writing‐original draft (lead); Writing‐review & editing (lead). **Ryoko Oka:** Data curation (lead); Formal analysis (supporting); Investigation (lead); Methodology (supporting); Validation (lead); Writing‐original draft (supporting). **Kaoru Baba:** Data curation (supporting); Formal analysis (supporting); Investigation (equal); Methodology (supporting); Resources (supporting); Validation (supporting). **Takahiro Noda:** Data curation (supporting); Formal analysis (supporting); Investigation (equal); Methodology (supporting); Validation (supporting). **Yuki Yoshida:** Formal analysis (lead); Investigation (supporting); Software (supporting); Validation (supporting); Visualization (supporting); Writing‐original draft (supporting); Writing‐review & editing (supporting). **Kenji Sato:** Data curation (supporting); Formal analysis (supporting); Investigation (supporting); Writing‐review & editing (supporting). **Kohei Kiyota:** Data curation (supporting); Investigation (supporting); Validation (supporting); Writing‐original draft (supporting). **Tohru Mizutare:** Data curation (supporting); Investigation (supporting); Methodology (supporting); Validation (supporting); Writing‐original draft (supporting). **Ryu Yoshida:** Conceptualization (supporting); Investigation (supporting); Methodology (supporting); Resources (supporting); Supervision (supporting); Writing‐original draft (supporting). **Akihiko Sato:** Conceptualization (supporting); Methodology (supporting); Project administration (supporting); Resources (supporting); Supervision (supporting); Writing‐original draft (supporting). **Hiroshi Kamimori:** Data curation (supporting); Formal analysis (supporting); Methodology (supporting); Project administration (supporting); Resources (supporting); Supervision (supporting). **Takao Shishido:** Conceptualization (equal); Investigation (supporting); Methodology (supporting); Resources (supporting); Supervision (supporting); Validation (supporting); Writing‐original draft (supporting); Writing‐review & editing (supporting). **Akira Naito:** Data curation (supporting); Formal analysis (supporting); Funding acquisition (supporting); Investigation (supporting); Methodology (supporting); Resources (lead); Supervision (supporting); Writing‐original draft (supporting); Writing‐review & editing (supporting).

## Supporting information

Supplementary Material

## Data Availability

All relevant data are included in the manuscript, including Supporting Information. Data sharing is not applicable to this study.
