# Supplementary material for: The antiviral effects of baloxavir marboxil against influenza A virus infection in ferrets
Source: Influenza Other Respir Viruses. 2020 Jun 13;14(6):710–9. doi: 10.1111/irv.12760 (PMC7578299; doi:10.1111/irv.12760)
Supplement: Supplementary file 1 — Supplementary Material [file IRV-14-710-s001.docx]

**Supporting information for the manuscript**

The antiviral effects of baloxavir marboxil against influenza A virus infection in ferrets

Mitsutaka Kitano^1^, Takanobu Matsuzaki^1^, Ryoko Oka^1^, Kaoru Baba^2^, Takahiro Noda^2^, Yuki Yoshida^1^, Kenji Sato^1^, Kohei Kiyota^1^, Tohru Mizutare^1^, Ryu Yoshida^1^, Akihiko Sato^1^, Hiroshi Kamimori^1^, Takao Shishido^1^, and Akira Naito^1^

^1^Shionogi & Co., Ltd., Toyonaka, Osaka, 561-0825, Japan

^2^Shionogi TechnoAdvance Research, Co., Ltd., Toyonaka, Osaka, 561-0825, Japan

**TABLE S-1 Pharmacokinetic parameters of baloxavir acid (BXA) in ferrets after a single oral administration of baloxavir marboxil (BXM) 10 mg/kg**

| BXA Parameters | | 0.5% MC | | | 5% SDS water | | | Ratio to 0.5% MC |
| --- | --- | --- | --- | --- | --- | --- | --- | --- |
| C_max_ | (ng/mL) | 15.8 | ± | 4.31 | 66.6 | ± | 14.6 | 4.2 |
| C_24hr_ | (ng/mL) | 0.223 | ± | 0.386 | 3.03 | ± | 2.33 | 13.6 |
| AUC_0-24hr_ | (ng·hr/mL) | 60.2 | ± | 25.2 | 421 | ± | 119 | 7.0 |
| T_max_ | (hr) | 0.50 | ± | 0.00 | 1.50 | ± | 0.58 | 3.0 |
| T_1/2_ | (hr) | 4.60 | ± | 2.94 | 6.91 | ± | 3.79 | 1.5 |

Data are expressed as the mean ± standard deviation of three ferrets (0.5% MC) or four ferrets (5% SDS water).

0.5% MC, 0.5% (w/v) methylcellulose; 5% SDS water, water containing 5% (w/v) sodium dodecyl sulfate (SDS) and 10% (w/w) polysorbate 80; C_max_, maximum concentration; C_24hr_, concentration at 24 hours; AUC_0-24hr_, area under the concentration-time curve from time 0 to 24 hours postdose; T_max_, time to maximum concentration; T_1/2_, terminal elimination half-life.

**TABLE S-2 Pharmacokinetic parameters of baloxavir acid (BXA) in ferrets after a single oral administration of baloxavir marboxil (BXM) 10 and 30 mg/kg (Individual data)**

| 10 mg/kg | | | | | | | | | | | | | | | | |
| --- | --- | --- | --- | --- | --- | --- | --- | --- | --- | --- | --- | --- | --- | --- | --- | --- |
| BXA Parameters | | | Ferret-01 | | Ferret-02 | | Ferret-03 | | Ferret-04 | | Mean | | ± | | S.D. | |
| C_max_ | (ng/mL) | | 59.4 | | 80.1 | | 77.3 | | 49.5 | | 66.6 | | ± | | 14.6 | |
| C_24hr_ | (ng/mL) | | 0.792 | | 3.90 | | 1.52 | | 5.90 | | 3.03 | | ± | | 2.33 | |
| AUC_0-24hr_ | (ng·hr/mL) | | 292 | | 576 | | 439 | | 378 | | 421 | | ± | | 119 | |
| T_max_ | (hr) | | 1.00 | | 2.00 | | 1.00 | | 2.00 | | 1.50 | | ± | | 0.58 | |
| T_1/2_ | (hr) | | 4.21 | | 6.47 | | 4.57 | | 12.4 | | 6.91 | | ± | | 3.79 | |
| BXM Parameters | | | Ferret-01 | | Ferret-02 | | Ferret-03 | | Ferret-04 | | Mean | | ± | | S.D. | |
| C_max_ | (ng/mL) | | 101 | | 79.2 | | 48.6 | | 431 | | 165 | | ± | | 179 | |
| C_24hr_ | (ng/mL) | | 3.59 | | 35.0 | | 6.75 | | 69.0 | | 28.6 | | ± | | 30.4 | |
| AUC_0-24hr_ | (ng·hr/mL) | | 174 | | 539 | | 318 | | 1830 | | 715 | | ± | | 758 | |
| T_max_ | (hr) | | 1.00 | | 0.500 | | 1.00 | | 2.00 | | 1.13 | | ± | | 0.63 | |
| T_1/2_ | (hr) | | N.C. | | N.C. | | N.C. | | N.C. | | N.C. | | | | | |
| 30 mg/kg | | | | | | | | | | | | | | | | |
| BXA Parameters | | Ferret-05 | | Ferret-06 | | Ferret-07 | | Ferret-08 | | Mean | | ± | | S.D. | |  |
| C_max_ | (ng/mL) | | 754 | | 489 | | 128 | | 89.6 | | 365 | | ± | | 316 | |
| C_24hr_ | (ng/mL) | | 21.3 | | 12.3 | | 3.38 | | 2.64 | | 9.91 | | ± | | 8.77 | |
| AUC_0-24hr_ | (ng·hr/mL) | | 7580 | | 3840 | | 1040 | | 508 | | 3240 | | ± | | 3240 | |
| T_max_ | (hr) | | 1.00 | | 1.00 | | 4.00 | | 2.00 | | 2.00 | | ± | | 1.41 | |
| T_1/2_ | (hr) | | 3.90 | | 4.32 | | 4.12 | | 5.41 | | 4.44 | | ± | | 0.67 | |
| BXM Parameters | | | Ferret-05 | | Ferret-06 | | Ferret-07 | | Ferret-08 | | Mean | | ± | | S.D. | |
| C_max_ | (ng/mL) | | 392 | | 353 | | 214 | | 233 | | 298 | | ± | | 88 | |
| C_24hr_ | (ng/mL) | | 22.7 | | 48.5 | | 28.7 | | 44.3 | | 36.1 | | ± | | 12.3 | |
| AUC_0-24hr_ | (ng·hr/mL) | | 1450 | | 1200 | | 1110 | | 1290 | | 1260 | | ± | | 150 | |
| T_max_ | (hr) | | 1.00 | | 1.00 | | 0.500 | | 1.00 | | 0.875 | | ± | | 0.250 | |
| T_1/2_ | (hr) | | N.C. | | N.C. | | N.C. | | N.C. | | N.C. | | | | | |

Dosing vehicle was water containing 5% (w/v) sodium dodecyl sulfate (SDS) and 10% (w/w) polysorbate 80.

C_max_, maximum concentration; C_24hr_, concentration at 24 hours; AUC_0-24hr_, area under the concentration-time curve from time 0 to 24 hours postdose; T_max_, time to maximum concentration; T_1/2_, terminal elimination half-life. N.C., not calculated.

**TABLE S-3 Simulated plasma concentration of baloxavir acid (BXA) in ferrets after a single dose or two doses of baloxavir marboxil (BXM) 10 and 30 mg/kg**

| Simulated plasma concentration (ng/mL) | | | | | |
| --- | --- | --- | --- | --- | --- |
| Single dose | | | Two doses | | |
| Time (h) | 10 mg/kg | 30 mg/kg | Time (h) | 10 mg/kg | 30 mg/kg |
| 0 (Dosing) | 0 | 0 | 0 (Dosing) | 0 | 0 |
| ***0.5*** | ***33.2*** | ***135*** | ***0.5*** | ***33.2*** | ***135*** |
| ***1*** | ***56.1*** | ***338*** | ***1*** | ***56.1*** | ***338*** |
| 1.5 | 55.6 | 341 | 1.5 | 55.6 | 341 |
| ***2*** | ***55.1*** | ***343*** | ***2*** | ***55.1*** | ***343*** |
| 2.5 | 48.5 | 316 | 2.5 | 48.5 | 316 |
| 3 | 42.6 | 292 | 3 | 42.6 | 292 |
| 3.5 | 37.5 | 269 | 3.5 | 37.5 | 269 |
| ***4*** | ***33.0*** | ***248*** | ***4*** | ***33.0*** | ***248*** |
| 4.5 | 29.1 | 235.6 | 4.5 | 29.1 | 236 |
| 5 | 25.7 | 223.8 | 5 | 25.7 | 224 |
| 5.5 | 22.7 | 212.6 | 5.5 | 22.7 | 213 |
| ***6*** | ***20.0*** | ***202*** | ***6*** | ***20.0*** | ***202*** |
| 6.5 | 19.1 | 190.3 | 6.5 | 19.1 | 190 |
| 7 | 18.3 | 179.2 | 7 | 18.3 | 179 |
| 7.5 | 17.5 | 168.8 | 7.5 | 17.5 | 169 |
| ***8*** | ***16.7*** | ***159*** | ***8*** | ***16.7*** | ***159*** |
| 8.5 | 15.8 | 146 | 8.5 | 15.8 | 146 |
| 9 | 15.0 | 134 | 9 | 15.0 | 134 |
| 9.5 | 14.2 | 123 | 9.5 | 14.2 | 123 |
| 10 | 13.5 | 112 | 10 | 13.5 | 112 |
| 10.5 | 12.8 | 103 | 10.5 | 12.8 | 103 |
| 11 | 12.1 | 94.5 | 11 | 12.1 | 94.5 |
| 11.5 | 11.5 | 86.6 | 11.5 | 11.5 | 86.6 |
| 12 | 10.9 | 79.4 | 12 (dosing) | 10.9 | 79.4 |
| 12.5 | 10.3 | 72.8 | 12.5 | 43.5 | 208 |
| 13 | 9.80 | 66.8 | 13 | 65.9 | 405 |
| 13.5 | 9.29 | 61.2 | 13.5 | 64.9 | 402 |
| 14 | 8.81 | 56.2 | 14 | 63.9 | 399 |
| 14.5 | 8.35 | 51.5 | 14.5 | 56.8 | 368 |
| 15 | 7.91 | 47.2 | 15 | 50.6 | 339 |
| 15.5 | 7.50 | 43.3 | 15.5 | 45.0 | 312 |
| 16 | 7.11 | 39.7 | 16 | 40.1 | 288 |
| 16.5 | 6.74 | 36.4 | 16.5 | 35.9 | 272 |
| 17 | 6.39 | 33.4 | 17 | 32.1 | 257 |
| 17.5 | 6.06 | 30.6 | 17.5 | 28.7 | 243 |
| 18 | 5.75 | 28.1 | 18 | 25.7 | 230 |
| 18.5 | 5.45 | 25.7 | 18.5 | 24.6 | 216 |
| 19 | 5.17 | 23.6 | 19 | 23.4 | 203 |
| 19.5 | 4.90 | 21.6 | 19.5 | 22.4 | 190 |
| 20 | 4.64 | 19.8 | 20 | 21.3 | 179 |
| 20.5 | 4.40 | 18.2 | 20.5 | 20.2 | 164 |
| 21 | 4.17 | 16.7 | 21 | 19.2 | 150 |
| 21.5 | 3.96 | 15.3 | 21.5 | 18.2 | 138 |
| 22 | 3.75 | 14.0 | 22 | 17.2 | 126 |
| 22.5 | 3.56 | 12.9 | 22.5 | 16.3 | 116 |
| 23 | 3.37 | 11.8 | 23 | 15.5 | 106 |
| 23.5 | 3.20 | 10.8 | 23.5 | 14.7 | 97.5 |
| ***24*** | ***3.03*** | ***9.91*** | 24 | 13.9 | 89.4 |
| 24.5 | 2.88 | 9.16 | 24.5 | 13.2 | 82.0 |
| 25 | 2.73 | 8.41 | 25 | 12.5 | 75.2 |
| 25.5 | 2.59 | 7.73 | 25.5 | 11.9 | 69.0 |
| 26 | 2.46 | 7.10 | 26 | 11.3 | 63.3 |
| 26.5 | 2.33 | 6.52 | 26.5 | 10.7 | 58.0 |
| 27 | 2.21 | 5.99 | 27 | 10.1 | 53.2 |
| 27.5 | 2.10 | 5.50 | 27.5 | 9.60 | 48.8 |
| 28 | 1.99 | 5.05 | 28 | 9.10 | 44.7 |
| 28.5 | 1.89 | 4.64 | 28.5 | 8.63 | 41.0 |
| 29 | 1.79 | 4.27 | 29 | 8.18 | 37.6 |
| 29.5 | 1.70 | 3.92 | 29.5 | 7.76 | 34.5 |
| 30 | 1.61 | 3.60 | 30 | 7.36 | 31.7 |
| 30.5 | 1.53 | 3.31 | 30.5 | 6.98 | 29.0 |
| 31 | 1.45 | 3.04 | 31 | 6.62 | 26.6 |
| 31.5 | 1.38 | 2.79 | 31.5 | 6.27 | 24.4 |
| 32 | 1.30 | 2.56 | 32 | 5.95 | 22.4 |
| 32.5 | 1.24 | 2.35 | 32.5 | 5.64 | 20.5 |
| 33 | 1.17 | 2.16 | 33 | 5.35 | 18.8 |
| 33.5 | 1.11 | 1.99 | 33.5 | 5.07 | 17.3 |
| 34 | 1.06 | 1.83 | 34 | 4.81 | 15.8 |
| 34.5 | 1.00 | 1.68 | 34.5 | 4.56 | 14.5 |
| 35 | 0.951 | 1.54 | 35 | 4.32 | 13.3 |
| 35.5 | 0.902 | 1.41 | 35.5 | 4.10 | 12.2 |
| 36 | 0.855 | 1.30 | 36 | 3.89 | 11.2 |
| 36.5 | 0.811 | 1.19 | 36.5 | 3.69 | 10.4 |
| 37 | 0.770 | 1.10 | 37 | 3.50 | 9.51 |
| 37.5 | 0.730 | 1.01 | 37.5 | 3.32 | 8.73 |
| 38 | 0.693 | 0.925 | 38 | 3.15 | 8.02 |
| 38.5 | 0.657 | 0.850 | 38.5 | 2.99 | 7.37 |
| 39 | 0.623 | 0.781 | 39 | 2.83 | 6.77 |
| 39.5 | 0.591 | 0.717 | 39.5 | 2.69 | 6.22 |
| 40 | 0.561 | 0.659 | 40 | 2.55 | 5.71 |
| 40.5 | 0.532 | 0.605 | 40.5 | 2.42 | 5.25 |
| 41 | 0.505 | 0.556 | 41 | 2.30 | 4.82 |
| 41.5 | 0.479 | 0.511 | 41.5 | 2.18 | 4.43 |
| 42 | 0.454 | 0.469 | 42 | 2.07 | 4.07 |
| 42.5 | 0.431 | 0.431 | 42.5 | 1.96 | 3.74 |
| 43 | 0.409 | 0.396 | 43 | 1.86 | 3.43 |
| 43.5 | 0.388 | 0.364 | 43.5 | 1.76 | 3.15 |
| 44 | 0.368 | 0.334 | 44 | 1.67 | 2.90 |
| 44.5 | 0.349 | 0.307 | 44.5 | 1.59 | 2.66 |
| 45 | 0.331 | 0.282 | 45 | 1.50 | 2.44 |
| 45.5 | 0.314 | 0.259 | 45.5 | 1.43 | 2.25 |
| 46 | 0.298 | 0.238 | 46 | 1.35 | 2.06 |
| 46.5 | 0.282 | 0.219 | 46.5 | 1.28 | 1.90 |
| 47 | 0.268 | 0.201 | 47 | 1.22 | 1.74 |
| 47.5 | 0.254 | 0.184 | 47.5 | 1.16 | 1.60 |
| 48 | 0.241 | 0.169 | 48 | 1.10 | 1.47 |

Shading indicates data representing the mean value of observed single dosing data for four ferrets.

The plasma BXA concentrations were estimated by computing the superposition from observed single dosing data in Phoenix WinNonlin Software^®^.

**TABLE S-4 Stability of baloxavir marboxil (BXM) in the plasma of human, ferret, rat, dog and monkey models (individual data)**

| Substance | Incubation time | % Residual in plasma Individual value | | | | | | | | | |
| --- | --- | --- | --- | --- | --- | --- | --- | --- | --- | --- | --- |
|  |  | Human | | Ferret | | Rat | | Dog | | Monkey | |
| BXM | 10 minutes | 92.7 | 91.8 | 94.0 | 95.4 | 0.0 | 0.0 | 97.0 | 86.3 | 81.5 | 84.4 |
|  | 20 minutes | 80.0 | 77.1 | 90.0 | 90.9 | 0.0 | 0.0 | 79.0 | 80.5 | 71.3 | 72.3 |
|  | 30 minutes | 65.3 | 62.6 | 77.3 | 77.5 | 0.0 | 0.0 | 74.9 | 76.3 | 56.3 | 54.7 |
| BXA^a^ | 10 minutes | 8.4 | 8.3 | 4.6 | 4.7 | 102.8 | 106.3 | 2.8 | 2.8 | 8.9 | 9.1 |
|  | 20 minutes | 22.4 | 22.4 | 13.8 | 14.1 | 102.2 | 105.9 | 8.1 | 8.2 | 23.5 | 24.6 |
|  | 30 minutes | 38.4 | 37.8 | 24.9 | 25.0 | 103.0 | 103.4 | 15.6 | 15.6 | 44.0 | 42.1 |

^a^ % Residual of baloxavir acid (BXA) was calculated from BXM concentration at 0 minutes as molar concentration.

**TABLE S-5 Stability of baloxavir marboxil (BXM), oseltamivir and cefcapene pivoxil in liver microsomes of human, ferret, rat, dog and monkey models (individual data)**

| Substance | Incubation time | % Residual in liver microsome Individual value | | | | | | | | | | | |
| --- | --- | --- | --- | --- | --- | --- | --- | --- | --- | --- | --- | --- | --- |
|  |  | Human | | Ferret | | Rat | | Dog | | Monkey | | Control (Microsome free) | |
| BXM | 10 minutes | 8.4 | 8.0 | 97.0 | 99.9 | 25.3 | 25.2 | 0.1 | 0.1 | 0.0 | 0.0 | 72.2 | 90.3 |
|  | 20 minutes | 0.3 | 0.2 | 98.3 | 97.9 | 4.9 | 4.4 | 0.1 | 0.1 | 0.0 | 0.0 | 81.2 | 82.1 |
|  | 30 minutes | 0.0 | 0.0 | 87.4 | 94.2 | 0.7 | 0.5 | 0.0 | 0.1 | 0.0 | 0.0 | 86.1 | 98.1 |
| Oseltamivir | 10 minutes | 81.1 | 98.6 | 125.2 | 87.8 | 111.5 | 110.0 | 116.2 | 115.4 | 67.3 | 79.8 | 111.5 | 101.3 |
|  | 20 minutes | 59.3 | 70.2 | 120.0 | 94.3 | 123.1 | 115.3 | 111.1 | 108.1 | 57.7 | 60.4 | 115.2 | 95.7 |
|  | 30 minutes | 41.5 | 57.8 | 122.7 | 95.2 | 115.3 | 119.3 | 105.0 | 116.0 | 41.8 | 48.5 | 115.3 | 107.2 |
| Cefcapene pivoxil | 10 minutes | 1.7 | 2.5 | 2.6 | 3.8 | 20.4 | 22.8 | 5.1 | 5.6 | 0.0 | 0.0 | 76.9 | 81.8 |
|  | 20 minutes | 0.0 | 0.1 | 0.0 | 0.1 | 1.6 | 1.7 | 0.2 | 0.2 | 0.0 | 0.0 | 56.4 | 79.0 |
|  | 30 minutes | 0.0 | 0.0 | 0.0 | 0.0 | 0.1 | 0.1 | 0.0 | 0.0 | 0.0 | 0.0 | 58.6 | 72.1 |

**TABLE S-6 Ratio of concentration (24 and 48 hours after first dosing) of baloxavir acid (BXA) to EC_90_ and target concentration after two oral administrations of baloxavir marboxil (BXM) at 10 and 30 mg/kg**

| Dose | 10 mg/kg | | 30 mg/kg | |
| --- | --- | --- | --- | --- |
| Time after first dosing | 24 hours | 48 hours | 24 hours | 48 hours |
| BXA Concentration (ng/mL)^a^ | 13.9 | 1.10 | 89.4 | 1.47 |
| Ratio to EC_90_^b^ | × 33.2 | × 2.6 | × 213 | × 3.5 |
| Ratio to Target concentration^c^ | × 2.0 | × 0.16 | × 13.0 | × 0.21 |

^a^ Concentrations of BXA at 24 hours and 48 hours after two doses of BXM were estimated using the NonParametric Superposition function in Phoenix WinNonlin software based on the mean plasma concentrations of BXA following single-dose administration of BXM.

^b^ EC_90_ value of BXA was 0.88 nM (=0.42 ng/mL) against influenza A/Kadoma/3/2006 strain.

^c^ Target concentration was 6.85 ng/mL.


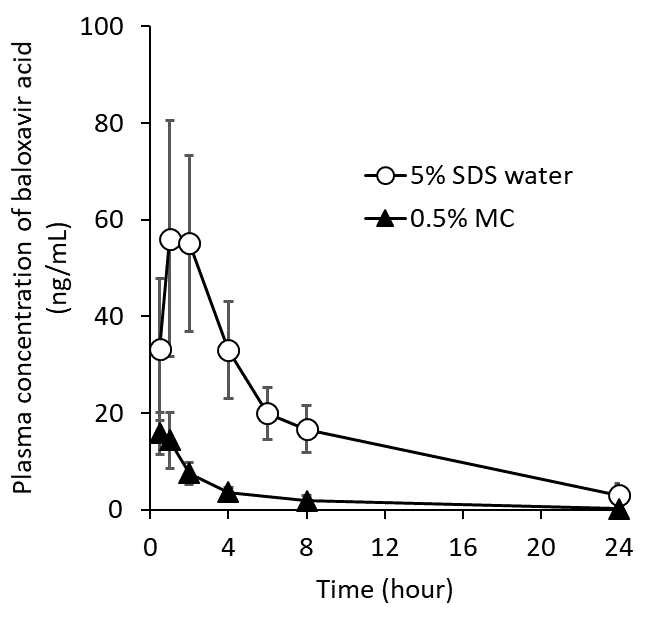


**FIGURE S-1 Vehicle effect on plasma concentration–time profiles of baloxavir acid (BXA) in ferrets after a single oral administration of baloxavir marboxil (BXM) 10 mg/kg**

Data are expressed as mean ± standard deviation for four ferrets (5% SDS water) or three ferrets (0.5% MC).

5% SDS water, water containing 5% (w/v) sodium dodecyl sulfate (SDS) and 10% (w/w) polysorbate 80; 0.5% MC, 0.5% (w/v) methylcellulose.


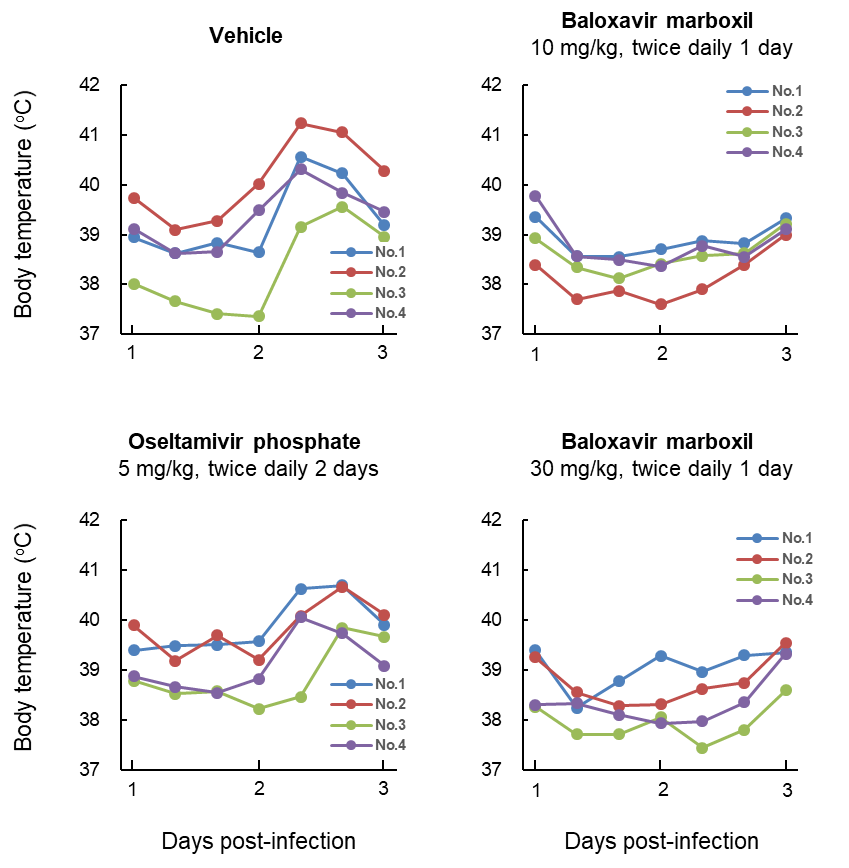


**FIGURE S-2 Body temperature of infected ferrets treated with vehicle, baloxavir marboxil (BXM) or oseltamivir phosphate (OSP), starting at Day 1 post-infection**

Ferrets were inoculated intranasally with A/Kadoma/3/2006 (1000 TCID_50_). Each plot represents the body temperature of individual ferrets treated with the investigative agent. Plot points represent the average temperature for an 8-hour period.

**
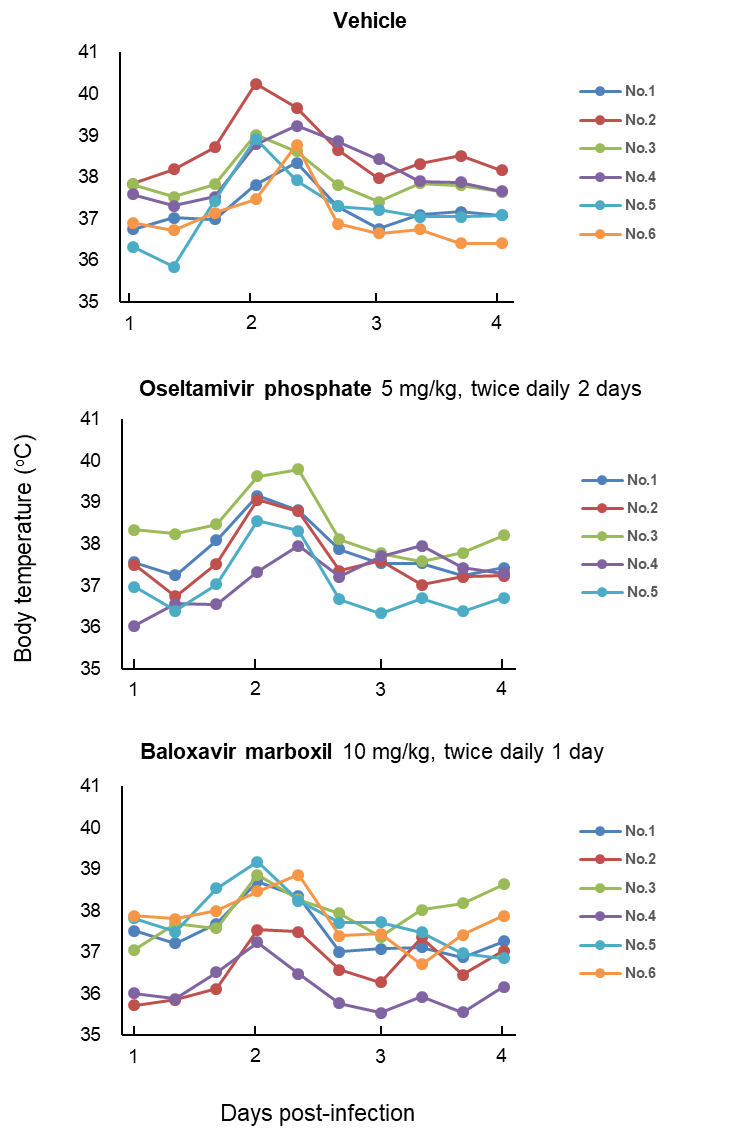
**

**Figure S-3 Body temperature of infected ferrets treated with vehicle, baloxavir marboxil (BXM) or oseltamivir phosphate (OSP), starting at Day 2 post-infection**

Ferrets were inoculated intranasally with A/Kadoma/3/2006 (5000 TCID_50_). Each plot represents the body temperature of individual ferrets treated with the investigative agent. Plot points represent the average temperature for an 8-hour period.


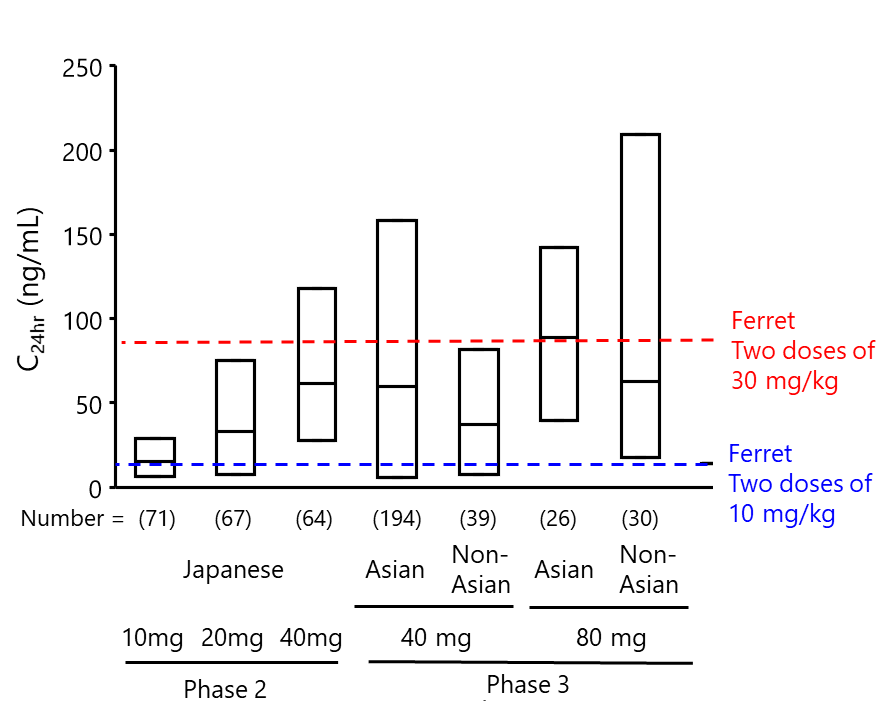


**Figure S-4 Plasma concentration of baloxavir acid (BXA) at 24 hours (C_24hr_) in humans and ferrets after dosing of baloxavir marboxil (BXM).** Box plot represents the mean and the range of BXA in clinical studies (data adapted from Koshimichi et al., J Pharm Sci 2019;108:1896–1904).
